# Editorial: Women in virology: 2023

**DOI:** 10.3389/fmicb.2024.1404214

**Published:** 2024-04-04

**Authors:** Ana Grande-Pérez, Asuka Nanbo, Na Jia, Antoinette Cornelia van der Kuyl

**Affiliations:** ^1^Área de Genética, Facultad de Ciencias, Instituto de Hortofruticultura Subtropical y Mediterránea “La Mayora” (IHSM-UMA-CSIC), Málaga, Spain; ^2^National Research Center for the Control and Prevention of Infectious Diseases, Nagasaki University, Nagasaki, Japan; ^3^State Key Laboratory of Pathogen and Biosecurity, Beijing Institute of Microbiology and Epidemiology, Beijing, China; ^4^Medical Microbiology and Infection Prevention, University of Amsterdam, Amsterdam, Netherlands

**Keywords:** women in science, female virologists, women in STEM, virology research, scientific equity

In the field of Virology, the contributions of women are as indispensable as they are diverse. From groundbreaking discoveries to innovative research methodologies, women have continually pushed the boundaries of virology, offering invaluable insights into the complex world of viruses. As we look back to 2023's research landscape, we not only celebrate the accomplishments of women in virology but also highlight the pressing need for gender equity in scientific research.

The Research Topic “*Women in virology: 2023*” encapsulates a mosaic of studies, each offering a unique perspective on critical aspects of virology. Among these contributions, one study stands out for its exploration of the intricacies of COVID-19 pathology. “*Comparison of plasma mitochondrial DNA copy number in asymptomatic and symptomatic COVID-19 patients*” by Shoraka et al., sheds light on a fundamental aspect of the disease, probing the potential links between mitochondrial DNA dynamics and COVID-19 symptomatology. This work not only enriches our understanding of COVID-19 pathogenesis but also underscores the contribution of female researchers in unraveling its complexities.

Furthermore, the emergence of SARS-CoV-2 variants has posed unprecedented challenges to global public health. In the midst of this ongoing battle, the study “*SARS-CoV-2 mutant spectra as variant of concern nurseries: endless variation?*” by Martínez-González et al., offers a critical examination of variant dynamics, exploring the notion of quasispecies as variant “nurseries” and their implications for viral evolution. By dissecting the genetic landscapes of SARS-CoV-2 mutants, this research provides a nuanced perspective on the evolutionary trajectories of the virus, further highlighting the substantial contributions of women to virological research.

Additionally, “*Controlling arbovirus infection: high-throughput transcriptome & proteome insights*” is a well-written and comprehensive review by Puig-Torrents and Díez that deepens into the realm of arbovirus infection control. Offering high-throughput transcriptome and proteome insights, this review promises to shape future strategies for combating these elusive pathogens. Puig-Torrents and Díez's thorough analysis provides valuable insights into the molecular mechanisms underlying arbovirus infection, paving the way for novel interventions.

The study titled “*The genetic variability and evolution of red-spotted grouper nervous necrosis virus quasispecies can be associated with its virulence*” by Ortega-del Campo et al., dives into the genetic variability and evolution of red-spotted grouper nervous necrosis virus quasispecies. Through meticulous analysis of whole genome quasispecies by Next Generation Sequencing, the authors shed light on the factors influencing nodavirus virulence offering key insights that can inform targeted interventions. Ortega-del Campo et al.'s work underscores the importance of understanding viral diversity in combating infectious diseases.

In addition, “*Raman-dielectrophoresis goes viral: towards a rapid and label-free platform for plant virus characterization*” by Sacco et al., presents an innovative approach to plant virus characterization. Through the integration of Raman spectroscopy and dielectrophoresis, Sacco et al. offer a rapid and label-free platform for characterizing plant viruses, revolutionizing the field of plant virology. Their pioneering work opens new avenues for the rapid detection and characterization of plant pathogens, with implications for agricultural sustainability and food security.

Lastly, “*Recombinant Newcastle disease viruses expressing immunological checkpoint inhibitors induce a pro-inflammatory state and enhance tumor-specific immune responses in two murine models of cancer*” by Santry et al., explores the intersection of virology and immunotherapy. Demonstrating the potential of recombinant Newcastle disease viruses in enhancing tumor-specific immune responses, the work of Santry et al. holds promise for the development of novel cancer therapeutics. By harnessing the power of viruses to modulate immune responses, this study offers a glimpse into the future of personalized cancer immunotherapy.

Beyond the specific findings of these studies, the Research Topic “*Women in virology: 2023*” serves as a testament to the remarkable diversity and ingenuity of women scientists in tackling some of the most pressing challenges in virology. From deciphering viral genomes to elucidating host-pathogen interactions, women researchers continue to drive innovation and discovery in the field of virology, shaping the future of infectious disease research side-by-side with their male peers.

As we pass the International Women's Day, let us not only celebrate the achievements of women in virology but also redouble our efforts to foster inclusivity and gender equity in scientific research. By championing the voices and contributions of women scientists, we not only enrich our understanding of viruses but also pave the way for a more equitable and diverse scientific community.

Join us as we explore this Research Topic through the lens of women researchers, and together, let us forge a path toward a brighter, more inclusive future for scientific discovery.

## Author contributions

AG-P: Writing – original draft, Writing – review & editing. AN: Writing – review & editing. NJ: Writing – review & editing. AvdK: Writing – review & editing.

